# Zeylenone, a naturally occurring cyclohexene oxide, inhibits proliferation and induces apoptosis in cervical carcinoma cells via PI3K/AKT/mTOR and MAPK/ERK pathways

**DOI:** 10.1038/s41598-017-01804-2

**Published:** 2017-05-10

**Authors:** Leilei Zhang, Xiaowei Huo, Yonghong Liao, Feifei Yang, Li Gao, Li Cao

**Affiliations:** Institute of Medicinal Plant Development, Chinese Academy of Medical Sciences and Peking Union Medical College, Beijing, 100193 China

## Abstract

There is a strong rationale to therapeutically target the PI3K/Akt/mTOR and MAPK/ERK pathways in cervical carcinoma since they are highly deregulated in this disease. Previous study by our group have demonstrated that Zeylenone (Zey) exhibited strong suppressive activity on PI3K/AKT/mTOR and MAPK/ERK signaling, providing a foundation to investigate its antitumor activity in cervical carcinoma. Herein, the present study aimed to investigate suppressive effect of Zey on HeLa and CaSki cells, and further explore the underlying mechanisms. Cells were treated with Zey for indicated time, followed by measuring its effects on cell viability, colony formation, cell cycle, cell apoptosis, and signal pathways. *In vivo* antitumor activity of Zey was then assessed with nude xenografts. We found that Zey substantially suppressed cell proliferation, induced cell cycle arrest, and increased cell apoptosis, accompanied by increased production of ROS, decreased mitochondrial membrane potential, activated caspase apoptotic cascade, and attenuated PI3K/Akt/mTOR and MAPK/ERK pathways. Additionally, *in vivo* experiments showed that Zey exerted good antitumor efficacy against HeLa bearing mice models via decreasing levels of p-PI3K and p-ERK. Collectively, these data clearly demonstrated the antitumor activity of Zey in cervical carcinoma cells, which is most likely via the regulation of PI3K/Akt/mTOR and MAPK/ERK pathways.

## Introduction

Cervical carcinoma remains the third most commonly diagnosed cancer and the fourth leading cause of mortality in females^1, 2^. Optimal treatment of early-stage cervical carcinoma includes surgery, radiation treatment, and cytotoxic chemotherapy^3, 4^. However, effective treatment options for advanced patients are limited^5, 6^. Human papilloma virus (HPV) is found to be associated with 99% of cervical carcinoma^7^, however, HPV infection alone is a necessary, but not sufficient, cause for the progression of invasive carcinoma, some other factors that promote proliferation and inhibit apoptosis could not be ignored in the long process of cervical carcinoma development^8–10^.

The phosphoinositide 3-kinase (PI3K)/Akt/mTOR and mitogen-activated protein kinase (MAPK)/extracellular signal-regulated kinase (ERK) oncogenic signaling pathways appeal much attention as they are frequently hyperactivated in cancer, deregulating control of metabolism, cell apoptosis, survival and proliferation^11, 12^. Excessive expression of PI3K/AKT/mTOR and MAPK/ERK signaling pathways can also be associated with altered sensitivity to targeted therapy when compared with patients that do not exhibit increased expression^13, 14^. These two pathways is generally actived by various mutations in human cancer occurring in upstream receptor genes such as EGFR, Flt-2, HER2, FMS, PDGFR, as well as chromosomal translocations (e.g., BCR-ABL). Accordingly, pharmacological agents that target these two pathways involved in cancer progression have been developed and are under clinical study, including drugs such as NPV-BEZ235, BKM120, Refametinib (BAY 86-9766), and Trametinib (GSK1120212)^15, 16^. Unfortunately, one inhibitor which targets one molecule in one pathway is likely to result in a compensatory activation of an additional oncogenic signaling pathway via an as yet undescribed mechanism, thereby diminishing the initial therapeutic effects of targeting either pathway alone^17–19^. To get around this problem, dual inhibition of the complementary signaling pathways has emerged as an important strategy, providing good therapeutic responses compared to individual treatment^19–21^. However, the high systemic toxicity remains a concern, limiting their clinical use^22^. Hence, it is essential to develop additional agents with unique activity against both PI3K/AKT/mTOR and MAPK/ERK pathways in cervical carcinoma.

Zeylenone, isolated from ethanol extract of the leaves of *Uvaria grandiflora* Roxb. of the family Annonaceae, is a naturally occurring cyclohexene oxide, which exhibited strong suppressive activity in several cancer cells, including acute lymphoblastic leukemia, breast, prostate and hepatocellular carcinoma, with less toxicity on normal cell lines^23^. Our previous study have proved that Zey could simultaneously inhibit PI3K/AKT/mTOR and MAPK/ERK pathways (Data not published), indicating its potent activity against cervical carcinoma. Nevertheless, the role of this compound in cervical carcinoma and the underlying molecular mechanisms requires further study.

The purpose of the current study is therefore to investigate the antitumor effects of Zey on cervical carcinoma cells both *in vitro* and *in vivo*, and further characterized the underlying mechanisms. Our data demonstrated that Zey induced substantial apoptosis of cervical carcinoma cells coincided with a corresponding reduction of cellular proliferation, which was associated with simultaneous inhibition of PI3K/AKT/mTOR and MAPK/ERK pathways. *In vivo* assays with HeLa xenografts model confirmed the antitumor effects of Zey and verify the abrogation of PI3K/AKT/mTOR and MAPK/ERK pathways by Zey treatment. Together, these data suggest that Zey could potentially improve the therapeutic outcome in cervical carcinoma.

## Results

### Zey inhibits poliferation in cervical carcinoma cells

**Figure 1:**
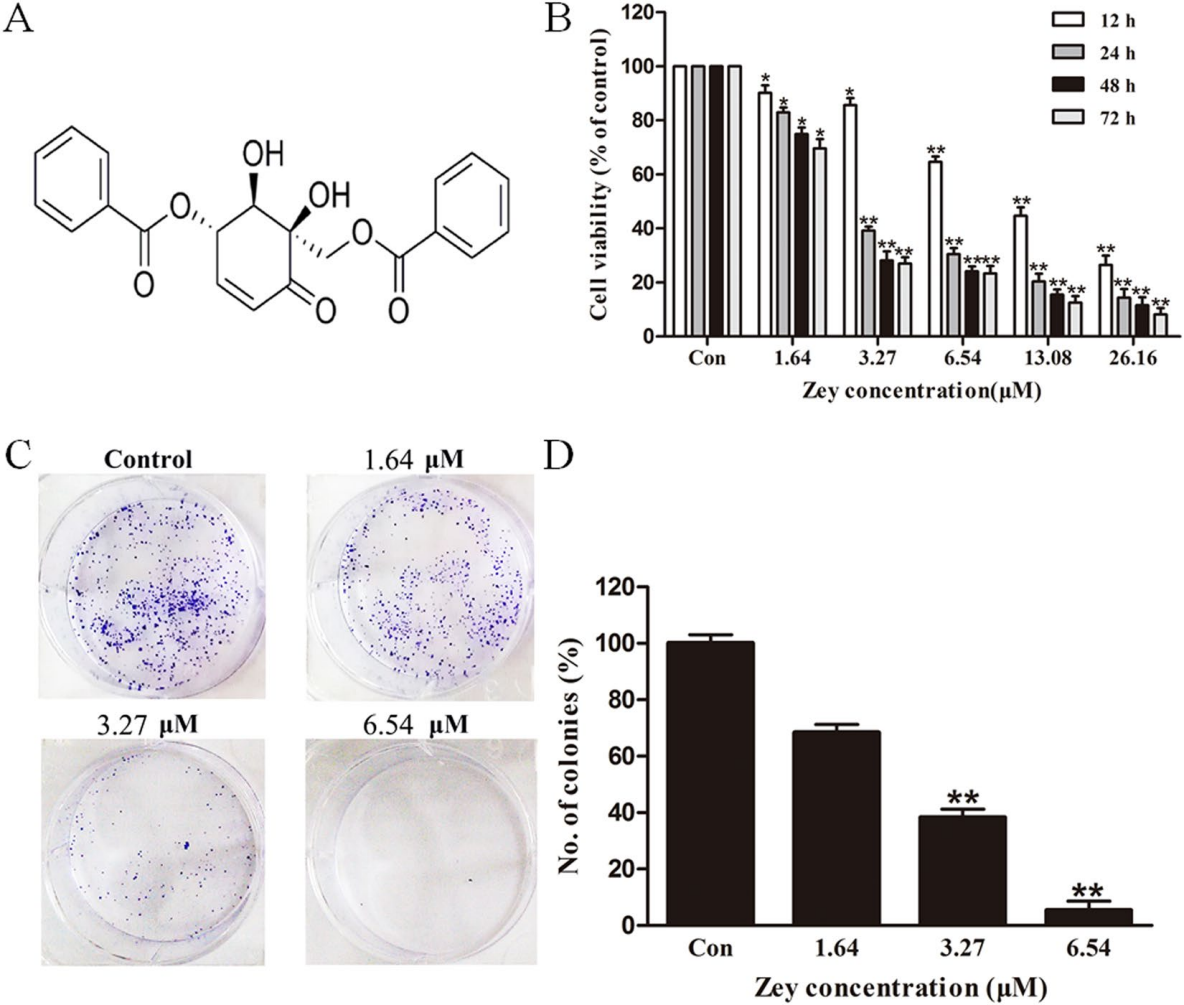
Zey effectively suppresses cell viability and colony formation in CaSki cells. (**A**) Chemical structure of Zey. (**B**) Cell viability determined by MTT assay. CaSki cells were treated with Zey (0, 1.64, 3.27, 6.54, 13.08, and 26.16 µM) for 12, 24, 48, and 72 h respectively. Data are expressed as means ± SD of 3 independent experiments. The cell viability of the Control (DMSO alone) is indicated as 100%. *P < 0.05, **P < 0.01 versus control cells. (**C**) Representative images of colonies after CaSki cells were treated with Zey for 14 days. (**D**) Statistical analysis of colony numbers from three independent experiments. *P < 0.05, **P < 0.01 versus control cells.

To evaluate the effect of Zey on the proliferation of cervical carcinoma cells, MTT assay was performed after cells were treated with various concentrations of Zey for 12 h, 24 h, 48 h, and 72 h, respectively. As shown in Fig. 1B and Supplementary Fig. S1, Zey treatments reduced the cell viability of the CaSki and HeLa cells in a dose- and time-dependent manner without severe toxicity to normal cells (Supplementary Table S1, Supplementary Table S2). IC_50_ values of Zey were detected to be 5.1, 3.3, 1.6, and 1.0 μM for CaSki cells and 6.1, 4.2, 2.1, and 1.4 μM for Hela cells, after cells were treated with Zey for 12 h, 24 h, 48 h, and 72 h, respectively, comparable to that of paclitaxel (Supplementary Table S3), indicating that Zey exhibits potent inhibitory activity on cervical carcinoma cells.

We next performed the colony formation assay to test the long-term effect of Zey on cervical carcinoma cells. Representative culture plates of HeLa and CaSki cells and the number of colonies were depicted in Fig. 1C,D, and Supplementary Fig. S2, respectively. These data revealed that HeLa and CaSki cells treated with Zey at indicated concentrations (HeLa: 0, 3.27, 6.54 and 13.08 μM; CaSki: 0, 1.64, 3.27 and 6.54 μM) for 24 h exhibited smaller and fewer colonies compared to untreated cells.

**Figure 2:**
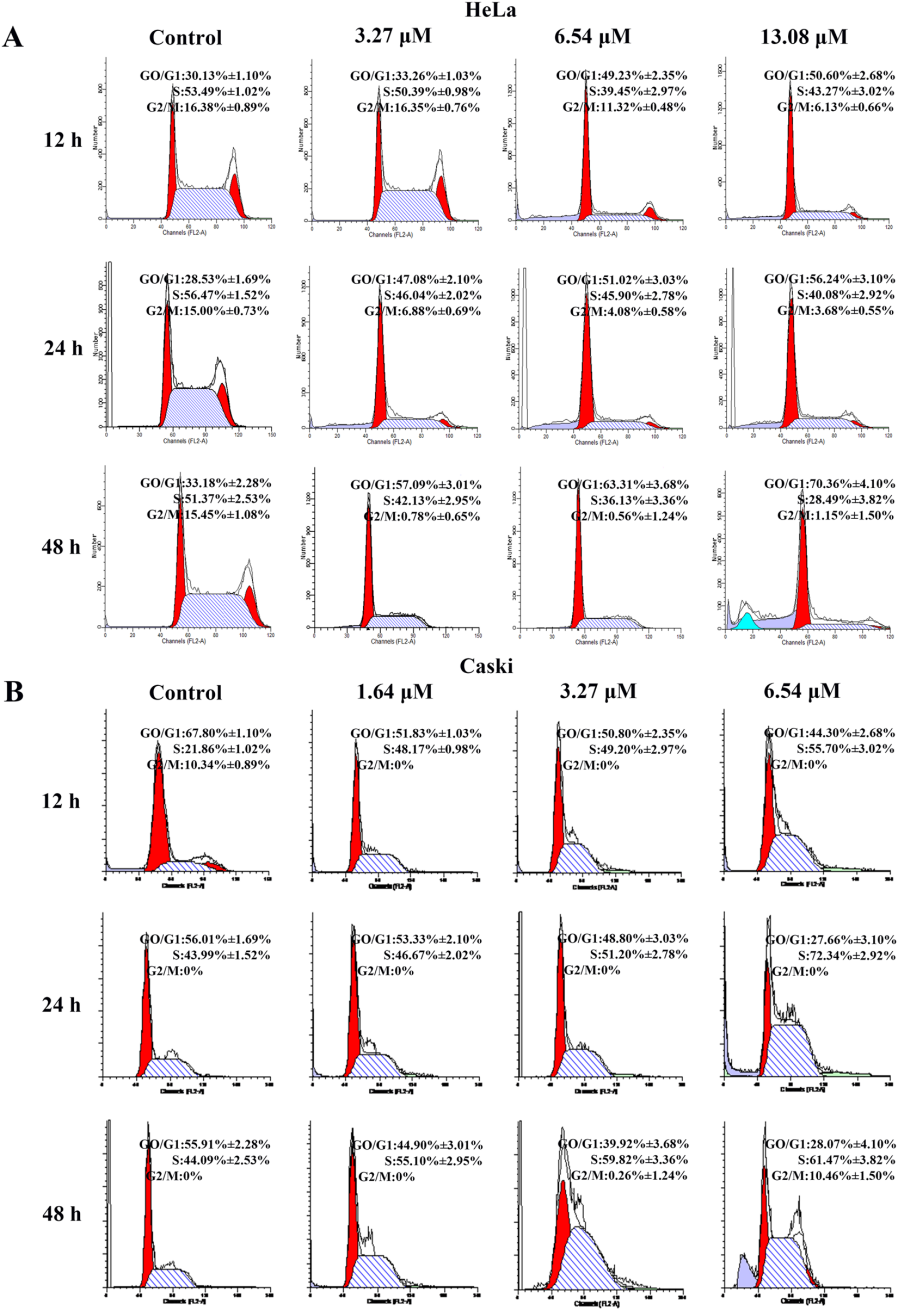
Zey induces cell cycle arrest in HeLa and CaSki cells. Cell cycle distributions of HeLa cells (**A**) and CaSki cells (**B**). HeLa and CaSki cells were treated with Zey for 12, 24, and 48 h, stained with propidium iodide, and then measured by flow cytometry.

Moreover, cell cycle assay was performed to further assess the effect of Zey on proliferation of cervical carcinoma cells. HeLa and CaSki cells were treated with Zey at different concentrations (HeLa: 0, 3.27, 6.54 and 13.08 μM; CaSki: 0, 1.64, 3.27 and 6.54 μM) for 12 h, 24 h, and 48 h, respectively, cell cycles were then detected by flow cytometry. Treatment of HeLa cells with different concentrations of Zey could dose- and time-dependently arrest cells at G_0_/G_1_ phase (Fig. 2A). Similarly, Zey treatment also induced cell cycle arrest in CaSki cells. As shown in Fig. 2B, treatment of CaSki cells with varying doses of Zey for 12 h, 24 h, and 48 h resulted in increased accumulation of cells in S phase as compared to untreated cells. The differential outcome of cell cycle arrest in these two cell lines might be because of different expression levels of proteins or receptors involving in regulation of cell cycle arrest in different cell lines^24–26^, which need further research.

### Zey induces apoptosis in cervical carcinoma cells

**Figure 3:**
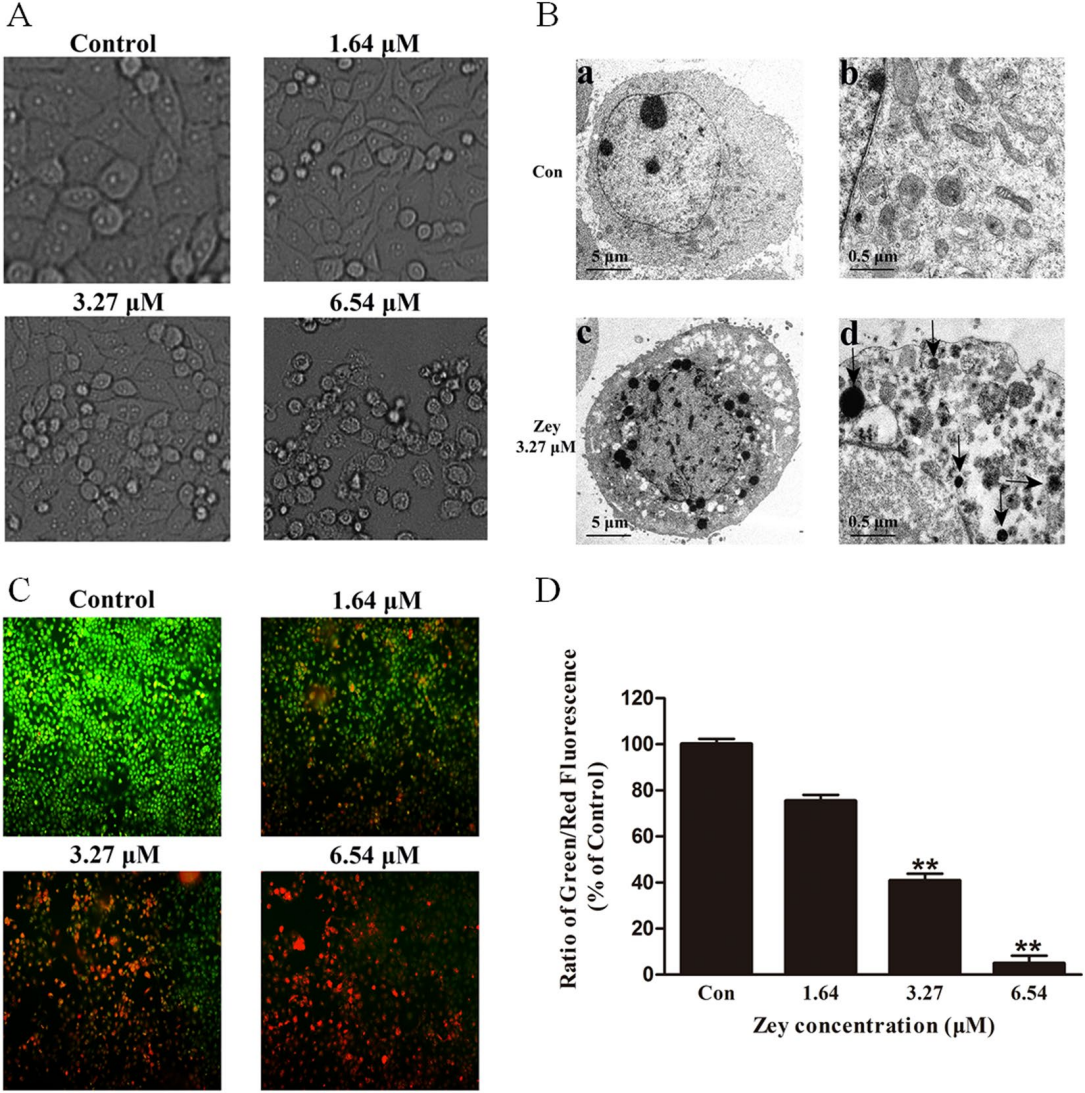
Zey induces apoptosis in CaSki cells. (**A**) Morphological changes of apoptosis observed by optical microscope. CaSki cells were treated with different concentrations of Zey (0, 1.64, 3.27, and 6.54 µM) for 24 h, and imaged using an Olympus digital camera. (**B**) Morphological changes of apoptosis observed by transmission electron microscopy. (a,b) Cells treated without Zey; (c,d) cells treated with Zey at 3.27 µM. The arrows indicate condensation and margination of nuclear chromatin surrounding in the nucleus. (**C**) Morphological observation with AO/EB double staining. (**D**) Statistical analysis of the Green/Red fluorescence ratios. **P < 0.01 versus control cells.

Several cell-based apoptosis assays were performed to determine whether the anti-cancer effect of Zey in cervical carcinoma cells was due to apoptosis. We first assessed morphological changes under microscope after HeLa and CaSki cells were treated with Zey for 24 h. As shown in Fig. 3A and Supplementary Fig. S3, a 24 h exposure of HeLa and CaSki cells to Zey resulted in a dose dependent increase of crushed cells, indicating that Zey induced cell death in HeLa and CaSki cells in a dose dependent manner. An apoptotic phenotype induced by Zey was further supported by condensation and margination of nuclear chromatin surrounding in the nucleus of HeLa and CaSki cells, which is regarded as indicator of apoptotic cell death (Fig. 3B and Supplementary Fig. S4).

AO/EB staining was simultaneously performed to investigate apoptotic cell death. As shown in Fig. 3C,D and Supplementary Fig. S5, a dose related increase of cells with orange nuclei (necrosis or terminal apoptosis cells) emerged after Zey treatment, while the untreated HeLa and CaSki cells displayed green nuclei (vigorously growing cells).

**Figure 4:**
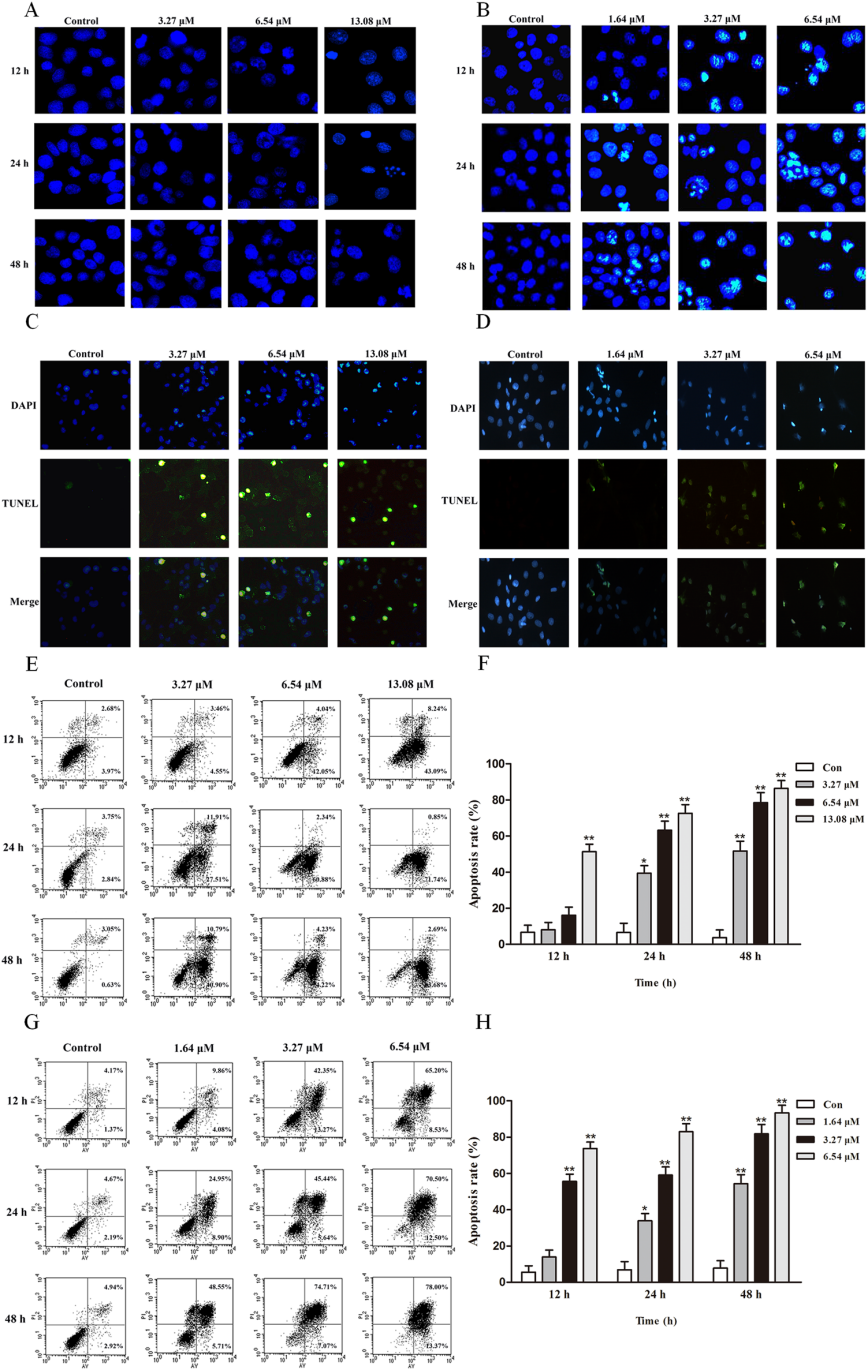
DNA segmentation of HeLa (**A**) and CaSki (**B**) cells detected by DAPI stain. TUNEL-assay for detection of apoptosis in HeLa (**C**) and CaSki (**D**) cells. (**E**,**F**) Apoptosis of HeLa cells detected by the Annexin V-FITC/PI staining test and the ratio of apoptosis cells. The data represented the means ± SD for triplicate determinations. *P < 0.05, **P < 0.01 versus control cells. (**G**,**H**) Apoptosis of CaSki cells detected by the Annexin V-FITC/PI staining test and the ratio of apoptosis cells, including early apoptotic cells (lower right quadrant) and late apoptotic cells (upper right quadrant). The data represented the means ± SD for triplicate determinations. *P < 0.05, **P < 0.01 versus control cells.

For a further assessment of apoptosis, DAPI, TUNEL, and Annexin V-FITC/PI assays were conducted. Initially, cervical carcinoma cells were stained with DAPI following exposure to different concentrations of Zey for 24 h. An increased number of cells with bright nuclear condensation or fragmented nuclei which was regarded as characteristics of cell apoptosis could be observed after Zey treatment (Fig. 4A,B). We next investigated apoptosis in cervical carcinoma cells using TUNEL assay. As shown in Fig. 4C,D, HeLa and CaSki cells treated with Zey were presented with a large proportion of apoptotic bodies. Additionally, rates of Zey induced apoptosis in HeLa and CaSki cells were assessed by Annexin-V/PI analysis. Cells undergoing early stage apoptosis (Annexin V-FITC positive, PI negative) and late stage apoptosis (both Annexin V-FITC and PI positive) were considered as apoptotic cells. The results showed that the apoptosis rates increased in a dose- and time- dependent manner in Zey-treated cells in comparison to untreated cells (Fig. 4E–H).

### Zey induces apoptosis in cervical carcinoma cells via the caspase apoptotic pathway

**Figure 5:**
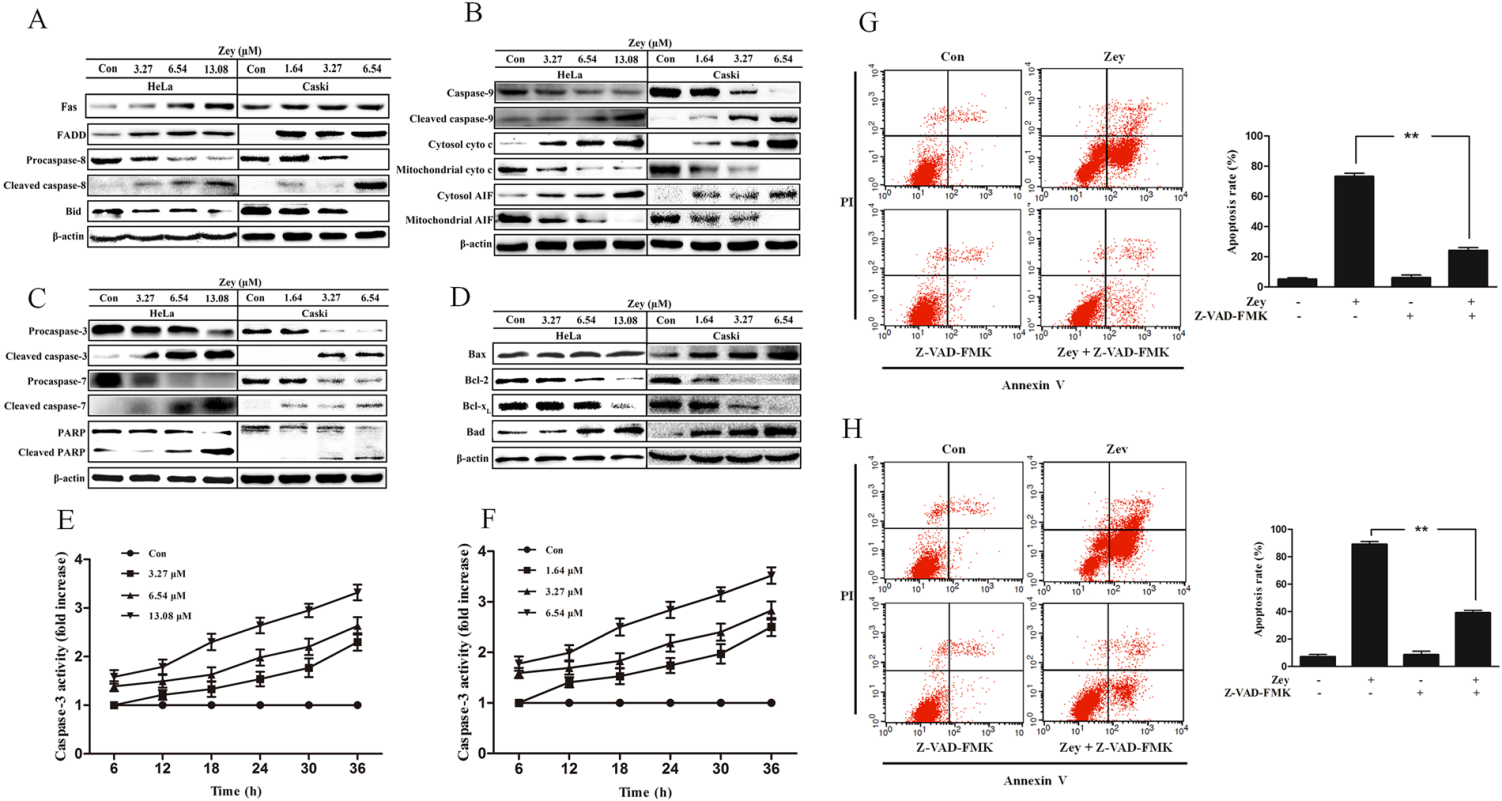
Zey induces apoptosis of HeLa and CaSki cells through caspase activation. (**A**) Zey induced expression changes of FAS, FADD, Bid, pro-caspase 8, and cleaved-caspase 8. (**B**) Zey induces release of AIF and cytochrome C from mitochondria and cleavage of caspase 9. HeLa and CaSki cells were treated with Zey for 24 h, then cell cytoplasm were extracted by digitonin buffer and the released AIF and cytochrome C were detected by western blot analysis. (**C**) Zey induces cleavage of PARP, caspases-3, and -7 in HeLa and CaSki cells. (**D**) Zey decreased expression of antiapoptotic proteins Bcl-XL, and Bcl-2, and increased expression of apoptotic proteins Bax and Bad. Zey inhibits caspase 3 activity of HeLa (**E**) and CaSki cells (**F**). Data were presented as the means ± SD for triplicate determinations. *P < 0.05, **P < 0.01 versus control cells. Pan caspase inhibitor Z-VAD-FMK significantly attenuated Zey-induced apoptosis in HeLa (**G**) and CaSki cells (**H**). Data are presented as the means ± SD for triplicate determinations. **P < 0.01 versus control cells.

To investigate the underlying mechanism involved in Zey-induced apoptosis in cervical carcinoma cells, the receptor mediated death pathway, also known as the extrinsic caspase pathway, was initially explored, as shown in Fig. 5A, Zey predominantly decreased expression of BID, pro-caspase-8 and markedly increased levels of FAS, FADD and cleaved caspase 8, indicating the involvement of extrinsic caspase pathway in Zey induced apoptosis. Moreover, Zey dose-dependently induced the cleavage of PARP which is regarded as an indicator of apoptosis, along with decreased expression of pro-caspases-3, -7, and -9, increased levels of the cleaved- caspases-3, -7, and -9, and increased release of cytochrome C and AIF from mitochondrial to the cytoplasm in Zey treated HeLa and CaSki cells (Fig. 5B,C), which indicates involvement of mitochondrial apoptosis pathway. Additional western blot analysis showed that Zey also markedly altered expression of Bcl-X_L_, Bad, and Bax, Bcl-2 in HeLa and CaSki cells (Fig. 5D).

Activation of caspase 3 were then detected in HeLa and in CaSki cells. The result revealed that Zey treatment dose- and time-dependently increased caspase 3 activity in both HeLa and in CaSki cells (Fig. 5E,F). To further confirm the involvement of caspase in Zey induced apoptosis, cells were pretreated with Z-VAD-FMK, a pan-caspase inhibitor, for 2 h. The result showed that pretreatment with Z-VAD-FMK completely abrogated apoptosis of HeLa and CaSki cells induced by Zey (Fig. 5G,H), indicating that apoptosis induced by Zey in HeLa and CaSki cells is tightly correlated with caspase.

**Figure 6:**
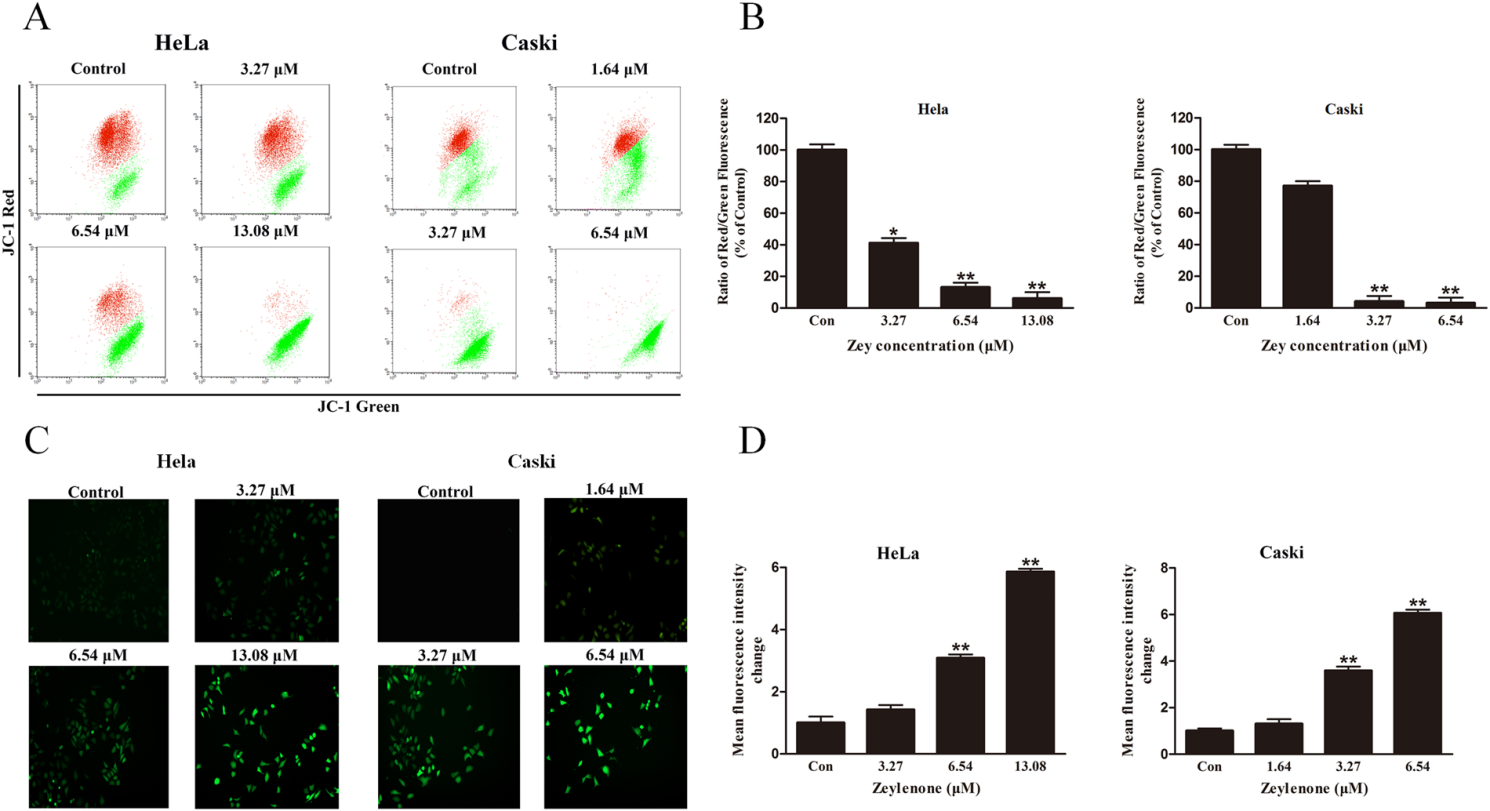
Zey induced mitochondrial dysfunction and ROS production. (**A**) Zey induced loss of mitochondrial membrane potential in HeLa and CaSki cells. Cells were treated with increasing concentrations of Zey for 24 h, mitochondrial membrane potential were analyzed after cells were stained with JC-1. (**B**) Quantitative analysis of the ratio of red to green fluorescence. Data were presented as the means ± SD for triplicate determinations. *P < 0.05, **P < 0.01 versus control cells. (**C**) ROS production was detected by fluorescence microscope after HeLa and CaSki cells were treated with Zey for 24 h. (**D**) Statistical analysis of fluorescence intensity after cells were stained with DCFH-DA.

Loss of ∆_Ψm_ triggers the release of cytochrome C and AIF from mitochondrial to the cytoplasm, which are potent activators of the apoptotic caspase cascade. We thus explored the effects of Zey on ∆_Ψm_. The results revealed that Zey treatment decreased ∆_Ψm_ in a dose dependent manner in both HeLa and CaSki cells (Fig. 6A,B), verifying involvement of mitochondria in Zey-induced apoptosis.

To further investigate the mechanism of Zey-induced apoptosis, we measured the production of reactive oxygen species (ROS) by DCFH-DA staining. The accumulation of fluorescent dye in HeLa and CaSki cells increased in a dose dependent manner with the treatment of Zey, as assessed by fluorescence microscopic and a microplate reader (Fig. 6C,D).

### Zey attenuates PI3K/AKT/mTOR and MAPK/ERK signaling pathways

**Figure 7:**
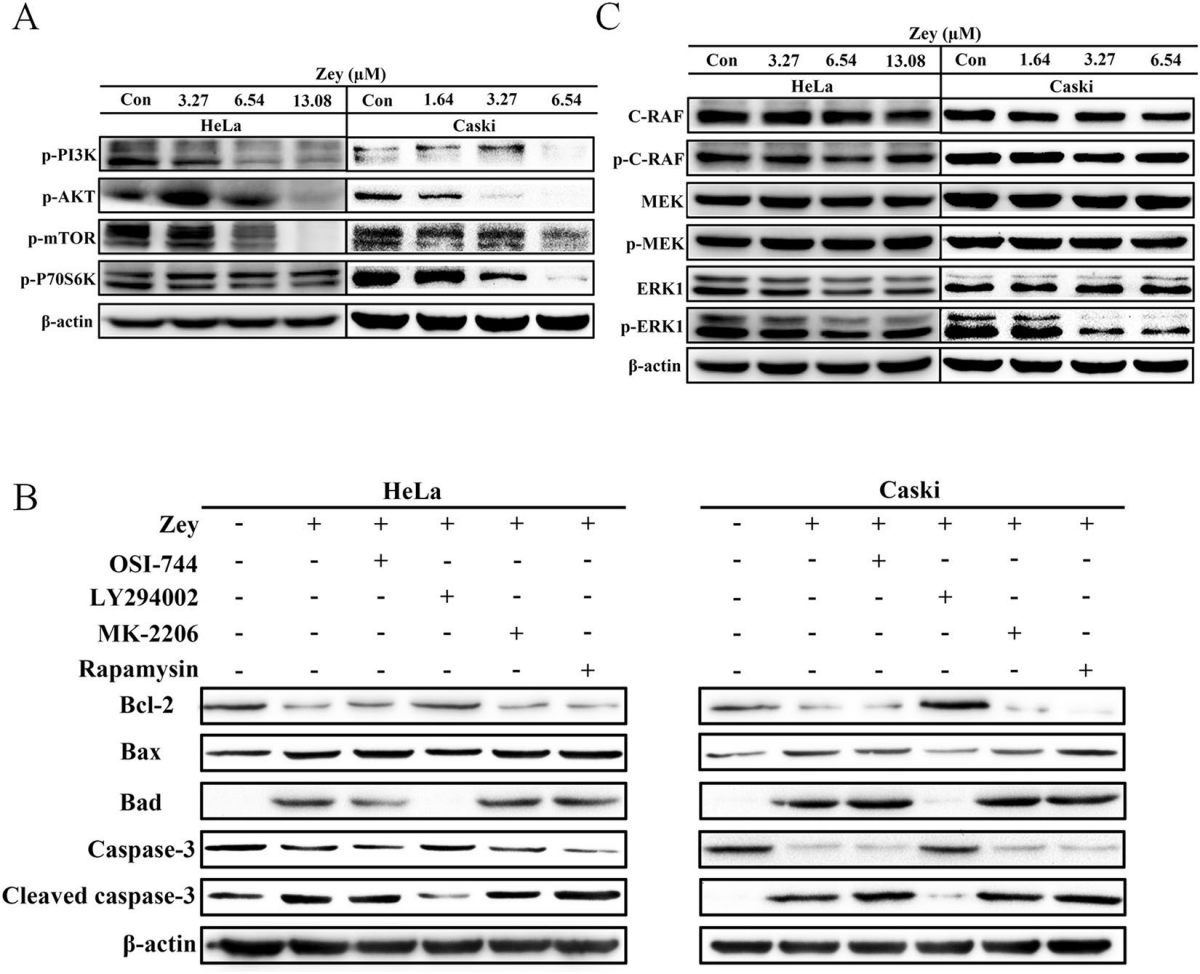
Zey attenuates PI3K/AKT/mTOR and MAPK/ERK pathways in HeLa and CaSki cells. (**A**) Immunoblot analyses of p-PI3K, p-AKT, p-mTOR and p-P70S6K in Zey-treated HeLa and CaSki cells. (**B**) Immunoblot analyses of apoptosis related proteins in HeLa and CaSki cells pretreated with different inhibitors. Cells were pretreated with EGFR inhibitor OSI-744, PI3K inhibitor LY294002, AKT inhibitor MK-2206, and mTOR inhibitor Rapamycin, respectively for 2 h, followed by Zey treatment for 24 h. (**C**) Immunoblot analyses of C-RAF, p-C-RAF, MEK, p-MEK, ERK, and p-ERK in Zey-treated HeLa and CaSki cells.

PI3K/AKT/mTOR and MAPK/ERK signaling pathways are reported to involved in malignant progress of cervical carcinoma. Initially, to test if the inhibitory activity of Zey on cervical carcinoma cells was associated with abrogation of PI3K/AKT/mTOR pathways, phosphorylation of PI3K, and corresponding signals AKT, mTOR, and P70S6K were detected by western blot analysis after cells were exposed to Zey for 24 h. As shown in Fig. 7A, Zey treatment strongly inhibited the phosphorylation of PI3K. Likewise, the phosphorylation levels of AKT, mTOR, and P70S6K were also effectively attenuated by Zey treatment.

To further confirm the apoptosis induced by Zey was associated with PI3K/AKT/mTOR signaling pathways, HeLa and CaSki cells were pretreated with EGFR inhibitor OSI-744, PI3K inhibitor LY294002, AKT inhibitor MK-2206, and mTOR inhibitor Rapamycin, respectively for 2 h, followed by evaluating the effect of Zey in these cells. Our results showed that decreased expression of Bcl-2 and caspase 3, and increased levels of Bax, Bad and cleaved-caspase 3 induced by Zey were abolished by pre-treatment with PI3K inhibitor LY294002, demonstrating the involvement of the PI3K/AKT/mTOR cascades, especially PI3K, in Zey induced apoptosis.

We further carried out experiments to investigated effects of Zey on MAPK/ERK pathway. As shown in Fig. 7C, Zey could predominantly inhibited phosphorylation of MEK and ERK. Collectively, these results indicated that the anti-tumor effect of Zey on HeLa and CaSki cells is tightly correlated with PI3K/AKT/mTOR and MAPK/ERK signaling pathways.

### Zey suppresses tumor growth in a mouse xenograft model

**Figure 8:**
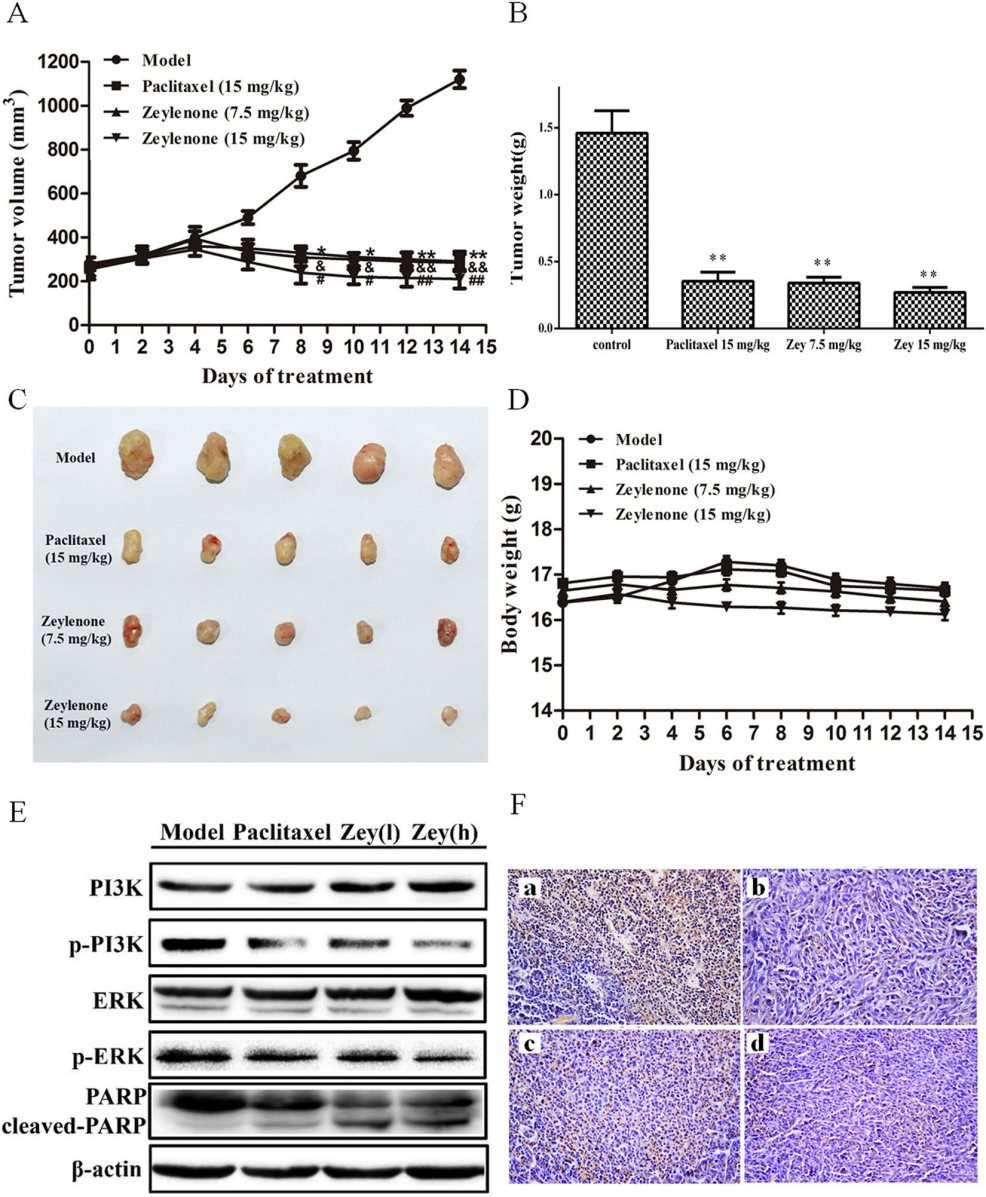
Zey inhibits tumor growth, induces clevage of PARP, and reduces expression of p-PI3K and p-ERK in nude BALB/c mice bearing HeLa xenograft. HeLa cells were transplanted subcutaneously to the BALB/c nude mice and subjected to Zey (7.5 and 15 mg/kg), paclitaxel (15 mg/kg) and saline (as the negative control) for 14 days. (**A**) Tumor volume of each group. (**B**) Tumor weight of each group. On day 14 after inoculation, the mice were sacrificed, and the tumor tissues were isolated, weighed, and summarized. Mean ± SD (n = 5). **P < 0.01, versus model group. (**C**) Photographs of tumors in each group. (**D**) Body weight were mesured every 2 days. (**E**) Immunoblot analyses of PI3K, p-PI3K, ERK, p-ERK, PARP, and cleaved-PARP in tumor tissues (**F**) Immunohistochemical analysis of p-PI3K in tumor tissues. (a) Mice treated with vehicles, (b) mice treated with Paclitaxel (15 mg/kg), (c) mice treated with Zeylenone (7.5 mg/kg), (d) mice treated with Zeylenone (15 mg/kg).

To further determine the antitumor activity of Zey *in vivo*, we used a xenograft model in which HeLa cells were subcutaneously injected under the flank of nude mice. As shown in Fig. 8A, Zey potently inhibited tumor growth as compared with the control group. The average weight of tumors from Zey treated mice was also significantly lower than that from the control mice (Fig. 8B,C). Moreover, no significant changes in body weight or adverse effect were observed in Zey-treated mice (Fig. 8D).

To further confirm whether Zey inhibits tumor growth via the PI3K/AKT/mTOR and MAPK/ERK pathways, the expression of p-PI3K, p-ERK and cleaved-PARP were identified in the isolated tumor tissues. Western blot analysis showed that Zey substantially decreased phosphorylation of PI3K and ERK, meanwhile, increased the cleavage of PARP compared with the control group (Fig. 8E). IHC analysis also confirmed that Zey treatment significantly decreased expression of p-PI3K (Fig. 8F). Collectively, these results demonstrated that Zey exhibited antitumor activity *in vivo* against HeLa cells in a mouse xenograft model through attenuating PI3K/AKT/mTOR and MAPK/ERK pathways.

## Discussion

Previously, we proved that the natural herbal product Zey attenuated PI3K/AKT/mTOR and MAPK/ERK pathways in K562 cells (Data not published), and therefore, it is reasonable to hypothesize that Zey may have potential in suppression of cervical carcinoma with aberrantly activate PI3K/AKT/mTOR and MAPK/ERK pathways. Herein, the antitumor effects of Zey in HeLa and CaSki cells and the underlying mechanisms were investigated. Our findings show, for the first time, that Zey treatment inhibited cell viability, suppressed colony formation, induced cell cycle arrest, and increased cell apoptosis in HeLa and CaSki cells via attenuating the PI3K/AKT/mTOR and MAPK/ERK pathways. Besides, antitumor activity of Zey in HeLa xenografts could be observed obviously, accompanied by strong inhibition of p-PI3K and p-ERK pathways. These findings indicate that the natural compound Zey may be a novel therapeutic approach to selectively target the PI3K/AKT/mTOR and MAPK/ERK pathways in cervical carcinoma cells, providing a therapeutic option in the treatment of cervical carcinoma.

It is well known that caspases, a family of cysteine proteases, are integral parts of the apoptotic pathway^27^. Their activation during apoptosis can involve death receptor pathway where Caspase-8, the initiator of this pathway, was activated by the TNF family of death receptors, or can involve the mitochondrial apoptosis pathway where mitochondria releases cytochrome C and AIF^28, 29^. Both pathways ultimately result in the cleavage and activation of the major downstream effector protease, caspase-3. Similar results were also observed in the current study where Zey-induced apoptosis was accompanied by production of ROS, loss of mitochondrial membrane potential, release of cytochrome c and AIF, activation of caspase 9, caspase 7, and caspase 3, and altered expression of apoptosis related Bcl-2 family proteins. Additionally, the receptor mediated death pathway was also demonstrated to involve in Zey-induced apoptosis, as indicated by decreased expression of BID, pro-caspase-8 and increased levels of FAS, FADD and cleaved caspase 8. Moreover, the activity of caspase-3 was also predominantly increased in HeLa and CaSki cells after Zey treatment. It implies that the caspase apoptotic pathway involves in Zey-induced apoptosis in cervical carcinoma.

The PI3K/AKT/mTOR pathway is dysregulated in a large proportion of human cancers, including cervical carcinoma, regulating the apoptotic response via its ability to interact with a number of key players in the apoptotic process^30^. On one hand, activation of this pathway can directly lead to phosphorylation of transcription factors that resulting in increased expression of anti-apoptotic members of the Bcl-2 family and decreased levels of pro-apoptotic proteins, thus inhibiting the release of cytochrome c from the mitochondria and the activation of the apoptotic caspase cascade^31–33^. On the other hand, activated Akt is capable of inactivate Foxo-3, thereby blocking augment expression of proteins related to the death receptor pathway^34^. Collectively, these events caused by the activated PI3K/AKT/mTOR pathway suppress apoptosis of cancer cells. Besides, integration of signals by this pathway assures cell cycle entry via regulating key molecules such as c-Myc, 27Kip1 and cyclin D1^14, 35, 36^. The inhibition of PI3K/AKT/mTOR pathway could, therefore, provide a suppressive measure against cancer. Consistently, we found that attenuation of the PI3K/AKT/mTOR pathway by Zey treatment leads to activation of both the intrinsic and the extrinsic caspase pathways. Moreover, western blot analysis with EGFR inhibitor OSI-744, PI3K inhibitor LY294002, AKT inhibitor MK-2206, and mTOR inhibitor Rapamycin revealed that apoptosis induced by Zey could be abrogated by PI3K inhibitor LY294002, indicating superior effect of Zey on PI3K than other proteins in this cascades. In addition, after Zey treatment, the cell cycles were arrest at G0/G1 and S phase in HeLa and CaSki cells, respectively, accompanied by abrogation of the PI3K/AKT/mTOR pathway. Overall, these data confirmed that induction of apoptosis and inhibition of proliferation in Zey treated HeLa and CaSki cells was attributed to abrogation of the PI3K/AKT/mTOR pathway.

In general, cross-talk between the PI3K/AKT/mTOR and MAPK/ERK pathways exists in many cancer cells^22, 37^. Consequently, PI3K/AKT/mTOR pathway abrogation lead to a compensatory activation of the MAPK/ERK signaling pathway^17^. Thus, co-inhibition of the PI3K/AKT/mTOR and MAPK/ERK cascades has become keen pharmaceutical objectives^38^. Actually, anti-cancer therapeutics targeting these two pathways are currently being evaluated in several ongoing clinical trials^39^. The results showed that combined inhibition of both the PI3K/AKT/mTOR and MAPK/ERK pathways elicited dramatic anti-tumor effects in many tumor types as compared to targeting either pathway alone^40, 41^, but at the cost of additional toxicity due to a small therapeutic index between normal and cancer cells. Thus, it is urgent to search for novel agents that targeting these two signaling pathways adequately. In this study, we found that Zey treatment decreased the expression of p-PI3K, p-AKT, p-mTOR, and p-ERK in HeLa and CaSki cells thereby indicating simultaneous inhibition of PI3K/AKT/mTOR and MAPK/ERK pathways. *In vivo* study with HeLa xenografts confirmed the antitumor activity of Zey via attenuating the PI3K and MAPK pathways.

It may be conclude that the natural product Zey could inhibit proliferation and induce apoptosis in cervical carcinoma cells via attenuating the PI3K and MAPK pathways, though other molecular mechanism cannot be exclude. Additionally, *in vivo* study confirmed that Zey significantly inhibited HeLa xenografts, the mechanism of which involved in abrogation of both PI3K/AKT/mTOR and MAPK/ERK pathways. Thus, this study might provide fundamental knowledge for understanding the antitumor activity of Zey in cervical carcinoma cells.

## Materials and Methods

### Reagents

Preparations of Zeylenone and mPEG-PLGA loaded zeylenone nano-micelles were described previously^42^. Zeylenone used for *in vitro* study was stored as 130 mM solutions in DMSO at −20 °C and further diluted to desired working concentration before each use. mPEG-PLGA loaded zeylenone nano-micelles used for *in vivo* study was stored in a dry container at room temperature. Dulbecco’s Modified Eagle Medium (DMEM) and fetal bovine serum (FBS) were purchased from Hyclone (Logan, Utah, USA). Antibodies against Bcl-2, Bcl-xL, Bax, Bad, Bid, β-actin and PARP antibodies, as well as all secondary antibodies were purchased from Santa Cruz Biotechnology (Santa Cruz, CA). Antibodies against cytochrome C, AIF, Fas, FADD, procaspase-3, cleaved procaspase-3, procaspase-7, cleaved procaspase-7, procaspase-8, cleaved procaspase-8, procaspase-9, cleaved procaspase-9, p-PI3K, p-AKT, p-mTOR, p-P70S6, C-RAF, p-C-RAF, MEK, p-MEK, ERK1 and p-ERK1 were purchased from Cell Signaling Technology (Danvers, MA). Methyl thiazolyl tetrazolium (MTT), 5,5′,6,6′-tetrachloro-1,1′,3,3′- tetraethyl-imidacarbocyanine iodide (JC-1), acridine orange (AO), and ethidium bromide (EB) were purchased from Sigma Aldrich Chemical (St. Louis, MO). Annexin V-FITC kit was obtained from Biosea Biotechnology Co. (Beijing, China). All inhibitors (OSI-744, LY294002,MK-2206, and Rapamycin) were purchased from Selleck Chemicals and ChemieTek (Shanghai, China).

### Cell lines and Cell Culture

Human cervical carcinoma cell lines HeLa and CaSki were obtained from Chinese Academy of Medical Sciences Basic Medicine Cell Center (Beijing, China). Human cervical epithelial cells were provided by Procell Life Science Co., Ltd (Wuhan, China). Cells were maintained in DMEM media supplemented with 10% fetal bovine serum (FBS), 100 U/mL penicillin, and 100 µg/mL streptomycin at 37 °C in a humidified incubator at 5% CO_2._

### Cell proliferation assay

Cells cultured in 96 well plates at a density of 6 × 10^3^ cells/ well were treated with various concentrations of Zey for 12 h, 24 h, 48 h, and 72 h, respectively, and cell viability was measured by MTT assay, as previously described^43^. After incubation for indicated time, 10 µL MTT reagent (5 mg/mL) was added to the media and allowed to incubate for another 4 h at 37 °C in an atmosphere of 5% CO_2_. The medium was then removed and 100 µL DMSO were added to dissolve MTT crystals. Cell viability was evaluated by reading the plates at an absorbance of 540 nm using a microplate reader.

### Colony formation assay

The anchorage-dependent colony formation assay was performed by seeding cells into 6-well plates at a low density (400–500 cells/well). Cells were treated with Zey at the indicated concentrations and were allowed to incubate in a humidified atmosphere with 5% CO_2_ at 37 °C for 14 days. Cell aggregates containing 40 or more cells were considered colonies. The colonies were stained with 0.1% Coomassie blue, which were then counted under a microscope.

### Observation of cell morphology by optical microscope

CaSki cells plated on 6-well plates were treated with different concentrations of Zey (0, 1.64, 3.27 and 6.54 µM) for 24 h. The cells were then observed using an optical inverted microscopy (Olympus, Tokyo, Japan) and images were captured.

### Observation by transmission electron microscopy

CaSki cells treated with Zey (0 and 3.27 µM) for 24 h were pre-fixed in 2.5% cacodylate-buffered glutaraldehyde for 2 h, post-fixed in 1% osmium tetroxide, which were then dehydrated in a graded series of alcohol, and embedded in epon. Sections were stained with uranyl acetate and lead citrate, followed by examined with a JEM-1400 transmission electron microscope (JEOL, Japan).

### Cell cycle assays

For cell cycle assays, HeLa and CaSki cells were treated with Zey at the indicated concentrations (HeLa: 0, 3.27, 6.54 and 13.08 µM; CaSki: 0, 1.64, 3.27 and 6.54 µM) for 12 h, 24 h and 48 h, respectively. Both the adherent and nonadherent cells were harvested, pooled, and fixed with 70% ethanol at −20 °C overnight. The cells were then stained with staining solution (50 µg/mL propidium iodide (PI) and 30 µg/mL RNase A in 1X PBS) and the percentage of cells in specific cell-cycle phases was determined (G1, S, and G2/M) using a FACSort flow cytometer (Becton-Dickinson, CA, USA).

### DAPI staining

After treated with different concentration of Zey (HeLa: 0, 3.27, 6.54 and 13.08 µM; CaSki: 0, 1.64, 3.27 and 6.54 µM), cells were collected and fixed in ice-cold 4% paraformaldehyde for 10 min at room temperature. The cells were then stained with 5 µg/mL of DAPI for 20 min at 37 °C and examined for fluorescence.

### AO/EB staining

CaSki cells were treated with different concentrations of Zey for 24 h. After washing with phosphate-buffered saline (PBS), cells were stained with AO/EB solution (containing 100 µg/mL AO and 100 µg/mL EB). Microscope images were then captured using a fluorescence microscope.

### JC-1 for mitochondrial transmembrane potential study

Cells were incubated in the presence of different concentrations of Zey (HeLa: 0, 3.27, 6.54 and 13.08 µM; CaSki: 0, 1.64, 3.27 and 6.54 µM) for 24 h and were then stained for 20 min with 10 µg/mL JC-1, which accumulates in mitochondria in a potential-dependent manner. The cells were collected, washed with PBS, and analyzed using a FACSort flow cytometer (Becton-Dickinson, CA, USA). Production of green fluorescence and reduction of the red fluorescence indicates loss of mitochondrial transmembrane potential.

### Annexin V-FITC/PI double-staining

Cells cultured in 6-well plates were treated with different concentrations of Zey (HeLa: 0, 3.27, 6.54 and 13.08 µM; CaSki: 0, 1.64, 3.27 and 6.54 µM) for 12 h, 24 h and 48 h, respectively. Both the adherent and nonadherent cells were harvested, pooled, and washed with PBS. Apoptosis was analyzed by double-staining with Annexin V-FITC and PI using an Annexin V-FITC apoptosis detection kit (BD Biosciences, San Jose, CA) according to the manufacturer’s recommended procedure. The stained cells (10^4^ cells) were then analyzed immediately using a FACS Calibur cytometer (Becton Dickinson, CA, USA) and results were expressed as percentage of living (AnnV−, PI−), early apoptotic (AnnV+, PI−), and late apoptotic/dead cells (AnnV+, PI+). Apoptotic rates were reported as the percentage of apoptotic cells among total cells.

### TUNEL apoptosis detection

Terminal deoxynucleotidyl transferase-mediated dUTP nick end labeling (TUNEL) assays were conducted to assess the presence and location of apoptosis, using a commercial kit (Millipore, Billerica, MA). Briefly, HeLa and CaSki cells were plated onto an 18-mm cover glass in media and cultured for 24 h before treated with Zey at different concentrations for 24 h. The cells were subsequently fixed in an acetic acid:ethanol (3:1) solution for 10 min at −20 °C and rinsed with PBS. Equilibration bufferand terminal deoxynucleotidyl transferase (TdT) enzyme were added, and the cover glass was incubated for 1 h at 37 °C. The cells were then incubated with anti-digoxigenin peroxidase conjugate and stained with peroxidase substrate, followed by mounted in Permount fluid and observed under a fluorescence microscope at 200× magnification.

### Reactive oxygen species (ROS) production

The cellular ROS levels were determined using total ROS detection kits according to the manufacturer’s brochures (Beyotime Biotechnology, Beijing, China). Briefly, following drug treatment, HeLa and CaSki cells were harvested and washed once with 1 × washing buffer, and then the cells were incubated with 100 µL of 5-(and-6)-carboxy-2′,7′-dichlorodihydrofluorescein diacetate (carboxy-H2DCFDA) (25 µM final concentration) in darkness at 37 °C for 30 min. Cellular DCF fluorescence intensity was determined using a microplate reader with an excitation wavelength of 495 nm and emission wavelength of 529 nm. The ROS level was expressed as a percentage of the control. Microscope images were also captured using a fluorescence microscope.

### Measurement of caspase-3 activity

HeLa and CaSki cells cultured in 6-well plates were treated with Zey at the indicated concentrations for 24 h. The activity of caspase-3 in cervical carcinoma cell culture supernatant was measured using a Caspase-3 activity assay kit (Nanjing Jiancheng, China), according to the manufacturer’s protocol.

### Tumor xenograft study

Six-week-old female Nude BALB/c mice purchased from Vital River Laboratory Animal Technology Co., Ltd. (Beijing, China) were maintained and treated according to protocols (SLXD-2015091523) approved by the Institutional Animal Care and Use Committee at the Institute of Medicinal Plant Development, Chinese Academy of Medical Sciences. Mice were inoculated subcutaneously with 1 × 10^7^ HeLa cells on the right flanks. Tumor growth was monitored daily, when the tumor volume reached approximately 200–300 mm^3^, the mice were randomized to four groups of 5 mice per group and were treated with vehicle (saline), mPEG-PLGA loaded zeylenone nano-micelles (containing zeylenone 7.5 or 15 mg/kg/d, i.p.), and paclitaxel (15 mg/kg/2d, i.p.) for a total of 14 days. Meanwhile, mouse body weight and tumor size was measured every 2 day, tumor volume (V) was calculated using the formula V = (L × W^2^)/2, where L is the largest diameter and W is the diameter perpendicular to width. Mice were then euthanized and tumor xenografts were excised, weighed, stored, and fixed. Additionally, IHC and western blotting analysis were performed to investigate the effects of Zey on protein expression of signaling pathways in tumor tissues.

### Immunohistochemical (IHC) analysis

Paraffin-embedded 3 µm tumor specimens from each group were deparaffinized, dehydrated, and treated with 3% hydrogen peroxide for 10 min to quench the endogenous peroxidase. After consecutive washing with PBS, slides were incubated with p-PI3K antibodies at a dilution of 1:100 at 4 °C overnight, followed by incubation with biotinylated secondary antibodies (1:100) for 1 hour at room temperature. Signals were then detected using Diaminobenzidine Substrate kit (Vector Laboratories). Images of the sections were captured and quantified using ImagePro-Plus software 6.0 (Media Cybernetics, Rockville, MD, USA).

### Western blot analysis

After the indicated treatments, cells were lysed with ice-cold RIPA buffer containing protease and phosphatase inhibitor cocktail purchased from Cowin Biotech (Beijing, China) for 30 min. Protein concentrations were determined using a BCA protein estimation kit (Cowin Biotech Co., Ltd., Beijing, China). Equal amount of proteins were separated by sodium dodecyl sulfate-polyacrylamide gel electrophoresis (SDS-PAGE) and transferred to PVDF membranes (Millipore Corporation, USA). The membranes were then incubated in blocking buffer containing 5% non-fat milk for 2 h and probed with primary antibody overnight at 4 °C. After washing with PBS, the membrane was incubated with horseradish peroxidase(HRP)-labeled secondary antibodies for 2 h at room temperature, washed with TBST, and then detected by enhanced chemiluminescence method using a commercial ECL kit (Cowin Biotech Co., Ltd.), according to manufacturer’s instructions. The levels of protein expression were quantified by image J software (National Institutes of Health, Bethesda, MD, USA) and normalized to the relative internal standards.

### Statistical analysis

All experiments were performed in triplicate, and data were expressed as Mean ± SD. GraphPad Prism 6.0 software (GraphPad Software) was used for statistical analysis. The statistical significance of group differences was analyzed with one-way ANOVA followed by Tukey’s test or Newman-Kueuls test. *P* < 0.05 was considered statistically significant.

## Electronic supplementary material

Zeylenone, a naturally occurring cyclohexene oxide, inhibits proliferation and induces apoptosis in cervical carcinoma cells via PI3K/AKT/mTOR and MAPK/ERK pathways
