# Supplementary material for: Zeylenone, a naturally occurring cyclohexene oxide, inhibits proliferation and induces apoptosis in cervical carcinoma cells via PI3K/AKT/mTOR and MAPK/ERK pathways
Source: Sci Rep. 2017 May 10;7:1669. doi: 10.1038/s41598-017-01804-2 (PMC5431878; doi:10.1038/s41598-017-01804-2)
Supplement: Supplementary file 1 — Zeylenone, a naturally occurring cyclohexene oxide, inhibits proliferation and induces apoptosis in cervical carcinoma cells via PI3K/AKT/mTOR and MAPK/ERK pathways [file 41598_2017_1804_MOESM1_ESM.pdf]

Zeylenone, a naturally occurring cyclohexene oxide, inhibits proliferation and induces apoptosis in cervical carcinoma cells via PI3K/AKT/mTOR and MAPK/ERK pathways

Leilei Zhang[1#], Xiaowei Huo[1#], Yonghong Liao[1], Feifei Yang[1], Li Gao[1], Li Cao[1*]

[1]Institute of Medicinal Plant Development, Chinese Academy of Medical Sciences and Peking Union Medical College, Beijing 100193, China

[#]Should be treated as the first author

Supplementary Table S1. Inhibitory effect of Zey on cancer cells after cells were treated with Zey for 48 h.

**Cell lines**	**IC50 (μM)**
Reh	2.76
RS4;11	0.36
HL-60	2.07
Jurkat	1.59
Molt-4	2.31
Astrocyte	7.52
Raji	2.22
U251	4.84
U87	5.05
U373	4.25
CHO	23.61
L02	24.33
PBMC	43.25

Supplementary Table S2. Inhibitory effect of Zey on normal human cervical epithelial cells (provided by Procell Life Science Co., Ltd.).

	24 h	48 h	72 h
IC50 (μM)	35.44	24.60	23.32

Supplementary Table S3. Inhibitory effect of paclitaxel on cervical cancer cells after cells were treated with paclitaxel for 48 h.

**Cell lines**	**IC50 (μM)**
HeLa	4.76
CasKi	4.06

Paclitaxel Injection was purchased from Beijing Union Pharm.

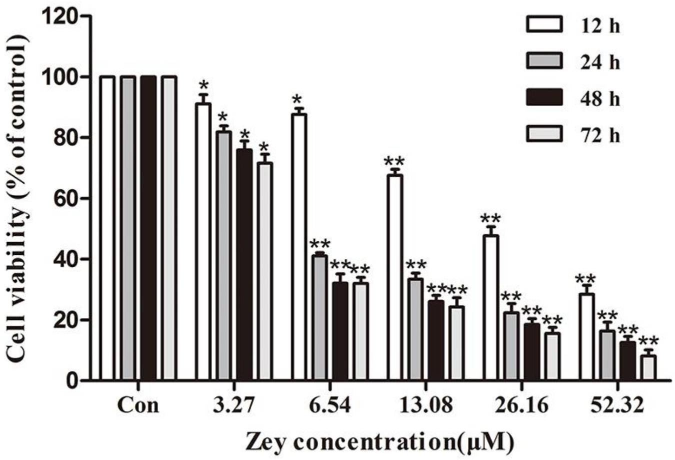

Supplementary Fig. S1. Zey effectively suppresses cell viability of HeLa cells. Cell viability determined by MTT assay. HeLa cells were treated with Zey (0, 3.27, 6.54, 13.08, 26.16, and 52.32 μM) for 12, 24, 48, and 72 h respectively. Data are expressed as means ± SD of 3 independent experiments. The cell viability of the Control (DMSO alone) is indicated as 100%. **P* < 0.05, ***P* < 0.01 versus control cells.

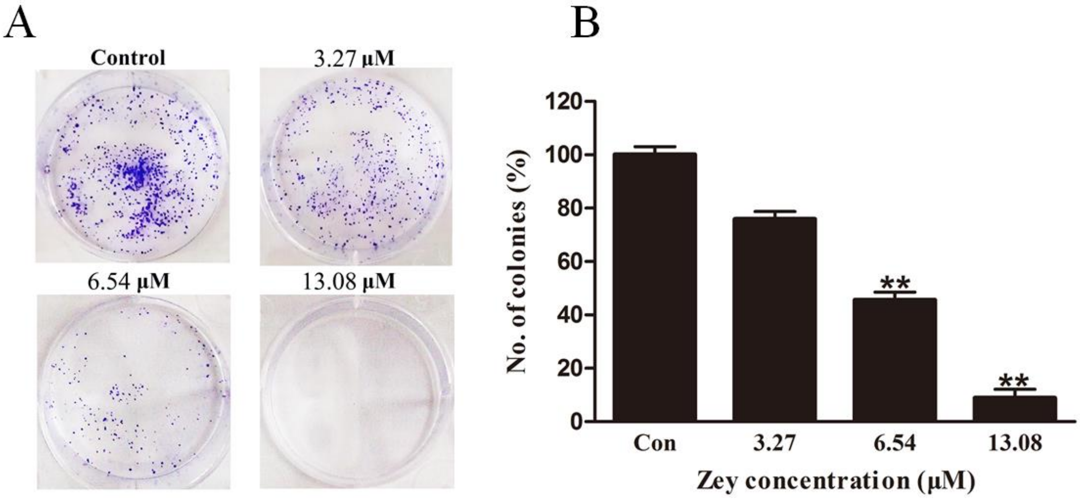

Supplementary Fig. S2. Zey effectively suppresses colony formation of HeLa cells. (A) Representative images of colonies after HeLa cells were treated with Zey for 14 days. (B) Statistical analysis of colony numbers from three independent experiments. **P* < 0.05, ***P* < 0.01 versus control cells.

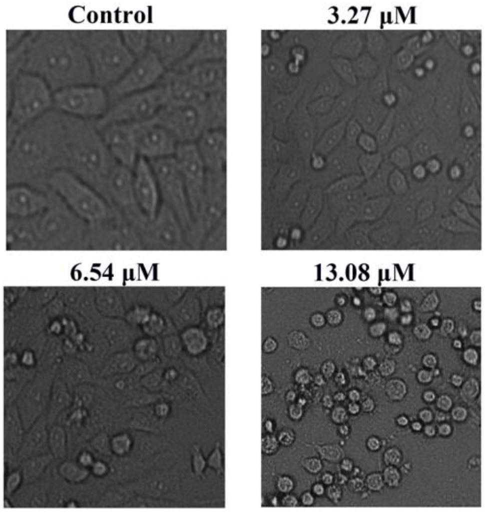

Supplementary Fig. S3. Morphological changes of apoptosis observed by optical microscope. HeLa cells were treated with different concentrations of Zey (0, 3.27, 6.54 and 13.08 μM) for 24 h, and imaged using an Olympus digital camera.

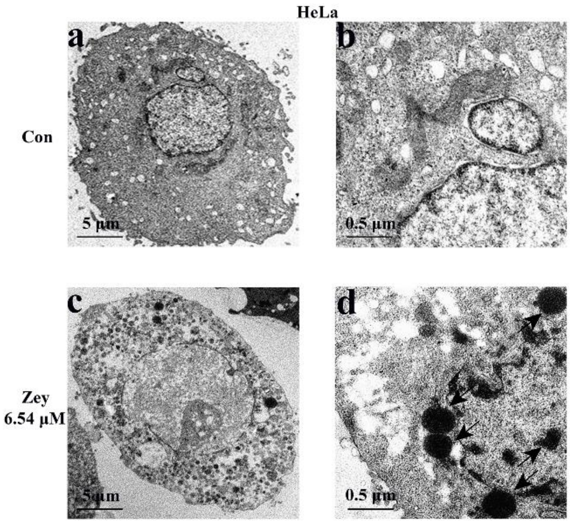

Supplementary Fig. S4. Morphological changes of apoptosis in HeLa cells observed by transmission electron microscopy. (a) and (b): cells treated without Zey; (c) and (d): cells treated with Zey at 6.54 μM. The arrows indicate condensation and margination of nuclear chromatin surrounding in the nucleus.

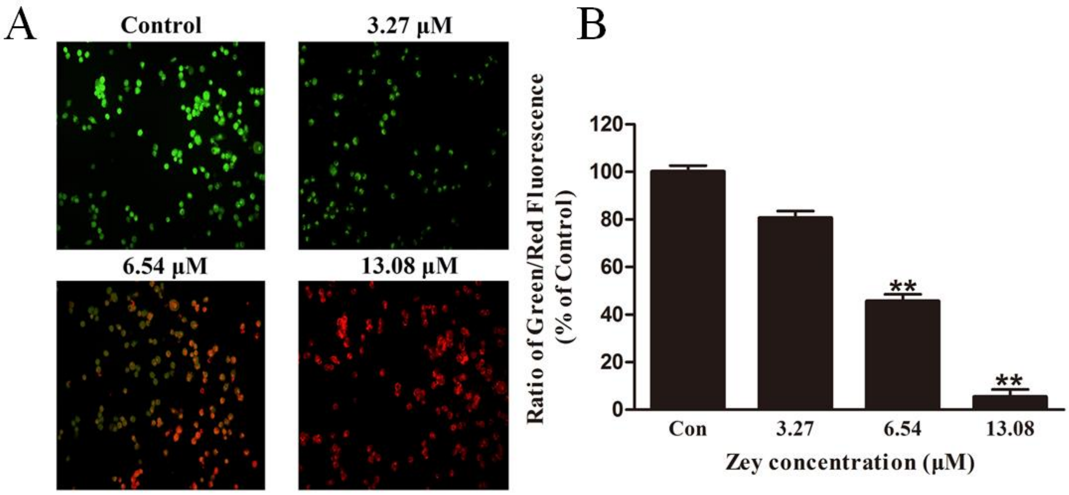

Supplementary Fig. S5. AO/EB staining in HeLa cells. (A) Morphological observation with AO/EB double staining. (B) Statistical analysis of the Green/Red fluorescence ratios. $^{**}P < 0.01$ versus control cells.

Report of Human Cell Line Authentication

I. Isolation

1. Tissue origin： Human cervical epithelium

2. Culture medium

Medium：M199

Supplementation：10% FBS; Insulin; bFGF; transfer; Penicillin; Streptomycin.

3. Methods

a. Isolated tissues under sterile conditions, and washed with PBS for three times；

b. Put the tissues into a centrifuge tube, crushed, and digested with mixed collagenase；

c. Centrifuged at 1000r/min for 5min, and then discarded the supernatant；

d. Cells were then suspended and cultured with M199 medium。

II. Authentication

1. Materials： Human cervical epithelium cells

Antibody:

Primary antibody：CK19

Secondary antibody：Goat Anti-Mouse IgG H&L -Cy3

Other regants： 4% paraformaldehyde, PBS

2. Methods：

a. Cells were grown on coverslips in a12-well plate at 37℃, 5% CO_2 for 24 h；

b. Fixed with 4% paraformaldehyde at room temperature for10 min；

c. Washed with PBS for three times and incubated with CK19 antibodies at 4 ℃ for 2 h；

d. Washed with PBS for three times and incubated with secondary antibodies at 37 ℃ for 1 h；

e. Washed with PBS for three times and then stained with hoechst 33258 for 15 min；

f. Washed with PBS for three times；

g. Images were captured using a fluorescence microscope.

III. Results

CK19: Red fluorescence represents CK19 positive, positive rates >90%

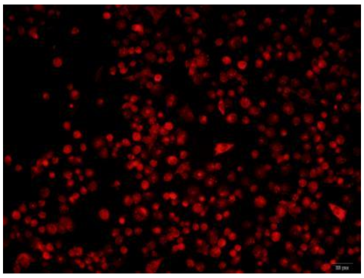

Figure 1-1 CK19

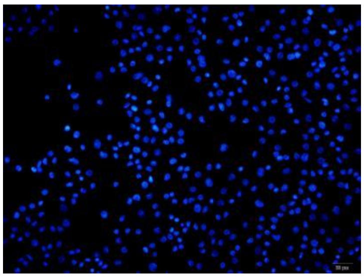

Figure 1-2 hoechst 33258

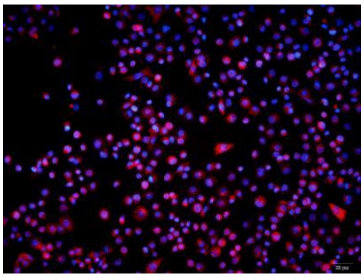

Figure 1-3 merge
